# Aberrant deposition of stress granule-resident proteins linked to *C9orf72*-associated TDP-43 proteinopathy

**DOI:** 10.1186/s13024-019-0310-z

**Published:** 2019-02-15

**Authors:** Jeannie Chew, Casey Cook, Tania F. Gendron, Karen Jansen-West, Giulia del Rosso, Lillian M. Daughrity, Monica Castanedes-Casey, Aishe Kurti, Jeannette N. Stankowski, Matthew D. Disney, Jeffrey D. Rothstein, Dennis W. Dickson, John D. Fryer, Yong-Jie Zhang, Leonard Petrucelli

**Affiliations:** 1 Department of Neuroscience, Mayo Clinic College of Medicine, 4500 San Pablo Rd, Jacksonville, FL 32224 USA; 2 Neurobiology of Disease Graduate Program, Mayo Clinic Graduate School of Biomedical Sciences, 4500 San Pablo Rd, Jacksonville, Florida, 32224 USA; 3 Department of Chemistry, The Scripps Research Institute, Scripps Florida, 130 Scripps Way #3A1, Jupiter, Florida, 33458 USA; 4 Department of Neurology, Brain Science Institute, Johns Hopkins University, 855 N Wolfe St, Baltimore, MD 21205 USA

**Keywords:** *C9orf72*, Stress granules, Frontotemporal dementia, Amyotrophic lateral sclerosis, TDP-43, Neurodegeneration

## Abstract

**Background:**

A G_4_C_2_ hexanucleotide repeat expansion in the noncoding region of *C9orf72* is the major genetic cause of frontotemporal dementia and amyotrophic lateral sclerosis (c9FTD/ALS). Putative disease mechanisms underlying c9FTD/ALS include toxicity from sense G_4_C_2_ and antisense G_2_C_4_ repeat-containing RNA, and from dipeptide repeat (DPR) proteins unconventionally translated from these RNA products.

**Methods:**

Intracerebroventricular injections with adeno-associated virus (AAV) encoding 2 or 149 G_4_C_2_ repeats were performed on postnatal day 0, followed by assessment of behavioral and neuropathological phenotypes.

**Results:**

Relative to control mice, gliosis and neurodegeneration accompanied by cognitive and motor deficits were observed in (G_4_C_2_)_149_ mice by 6 months of age. Recapitulating key pathological hallmarks, we also demonstrate that sense and antisense RNA foci, inclusions of poly(GA), poly(GP), poly(GR), poly(PR), and poly(PA) DPR proteins, and inclusions of endogenous phosphorylated TDP-43 (pTDP-43) developed in (G_4_C_2_)_149_ mice but not control (G_4_C_2_)_2_ mice. Notably, proteins that play a role in the regulation of stress granules – RNA-protein assemblies that form in response to translational inhibition and that have been implicated in c9FTD/ALS pathogenesis – were mislocalized in (G_4_C_2_)_149_ mice as early as 3 months of age. Specifically, we observed the abnormal deposition of stress granule components within inclusions immunopositive for poly(GR) and pTDP-43, as well as evidence of nucleocytoplasmic transport defects.

**Conclusions:**

Our in vivo model of c9FTD/ALS is the first to robustly recapitulate hallmark features derived from both sense and antisense *C9orf72* repeat-associated transcripts complete with neurodegeneration and behavioral impairments. More importantly, the early appearance of persistent pathological stress granules prior to significant pTDP-43 deposition implicates an aberrant stress granule response as a key disease mechanism driving TDP-43 proteinopathy in c9FTD/ALS.

**Electronic supplementary material:**

The online version of this article (10.1186/s13024-019-0310-z) contains supplementary material, which is available to authorized users.

## Background

A hexanucleotide repeat expansion consisting of hundreds to thousands of G_4_C_2_ repeats located in a non-coding intronic region of chromosome 9 open reading frame 72 (*C9orf72*) is the most frequent genetic cause of frontotemporal dementia (FTD) and amyotrophic lateral sclerosis (ALS), collectively referred to as c9FTD/ALS. FTD is second only to Alzheimer’s disease (AD) as a cause of dementia in patients under 65 [46], and encompasses a group of disorders characterized clinically by changes in personality, behavior, and/or language. ALS, a common motor neuron disease, is characterized by selective degeneration of lower and upper motor neurons, leading to muscle weakness, spasticity, and atrophy, and ultimately resulting in paralysis. Given that FTD and ALS patients often share clinical, genetic, and neuropathological features, including the aggregation of phosphorylated TAR DNA-binding protein 43 (pTDP-43) [47], common disease mechanisms are believed to underlie both disorders.

Potential mechanisms implicated in the pathogenesis of c9FTD/ALS include the loss of C9ORF72 protein function, repeat RNA-mediated toxicity, and toxicity from the accumulation of dipeptide repeat (DPR) proteins produced by repeat-associated non-ATG (RAN) translation (reviewed [19, 55, 56]). For instance, foci containing sense G_4_C_2_ or antisense G_2_C_4_ repeat RNA bidirectionally-transcribed from the *C9orf72* expansion are observed in c9FTD/ALS postmortem brain tissues, cultured cells, and neurons, and may disrupt RNA metabolism and nucleocytoplasmic transport through sequestration of various RNA-binding proteins [11, 14, 18, 22, 23, 31, 42, 43]. Moreover, RAN translation of repeat RNA produces poly-glycine-alanine (GA) and poly-glycine-arginine (GR) from sense G_4_C_2_ transcripts, poly-proline-alanine (PA) and poly-proline-arginine (PR) from antisense G_2_C_4_ transcripts, and poly-glycine-proline (GP) from both sense and antisense transcripts [2, 18, 43, 44, 65]. In postmortem c9FTD/ALS brains, poly(GA) and poly(GP) inclusions are more frequent than poly(GR), while poly(PA) and poly(PR) inclusions are comparatively rare [35, 36]. Although poly(GP) is believed to be relatively benign, poly(GA) deposition is linked to ubiquitin proteasome dysfunction, ER stress, and nucleocytoplasmic transport defects [21, 39, 62, 63]. In addition, despite being less abundant than poly(GA), several reports have demonstrated that poly(GR) and poly(PR) are particularly toxic in a variety of cell and animal models, leading to nucleolar stress and impairment of vital cellular functions including nucleocytoplasmic transport, stress granule (SG) dynamics, and protein translation [17, 25, 29, 41, 52, 54, 57, 61]. Nevertheless, how the many pathognomonic features of c9FTD/ALS cause disease remains to be determined, an effort that is hindered by the lack of a comprehensive model that recapitulates the hallmark features derived from both sense and antisense *C9orf72* repeat expansion transcripts, including TDP-43 pathology.

We previously described the development and characterization of a mouse model of c9FTD/ALS whereby the expression of (G_4_C_2_)_66_ throughout the central nervous system (CNS) by means of somatic brain transgenesis using an adeno-associated virus (AAV) vectors resulted in the development of *C9orf72*-associated pathological and behavioral abnormalities by 6 months of age [9]. Specifically, sense RNA foci, inclusions of poly(GP), poly(GA), and poly(GR), pTDP-43 pathology, gliosis, and neuronal loss were detected in the brain of (G_4_C_2_)_66_ mice [9]. In the present study, we describe the characterization of an AAV mouse model in which a longer repeat is expressed. Similarly to (G_4_C_2_)_66_ mice, G_4_C_2_-derived RNA foci and DPR proteins accumulate in (G_4_C_2_)_149_ mice, and they develop TDP-43 pathology, neurodegeneration, gliosis, and behavioral abnormalities. Most remarkably, however, was our finding that antisense G_2_C_4_ RNA-derived foci and DPR proteins were also present in (G_4_C_2_)_149_ mice, thus providing an in vivo model that develops all pathological features of c9FTD/ALS with which to explore disease mechanisms. Furthermore, we demonstrate that several SG-associated proteins localize to inclusions in (G_4_C_2_)_149_ mice, offering additional support to the growing body of evidence that aberrant regulation of SG biology contributes to c9FTD/ALS [4, 5, 27, 32, 37, 38, 59]. SGs are dynamic, membraneless structures that transiently form in the cytoplasm in response to various stressors, and subsequently dissolve upon stress resolution. However, chronic cellular stress and a persistent SG response have been proposed to mediate the formation of irreversible, pathological inclusions by providing an environment in which TDP-43 and other aggregation-prone proteins and RNAs are focally concentrated. In support of this idea, knockdown of SG components or inhibition of SG formation was recently shown to decrease c9FTD/ALS pathophysiology and reverse nucleocytoplasmic transport defects [59]. By revealing colocalization of poly(GR) and pTDP-43 with SG-resident proteins within inclusions combined with evidence of nucleocytoplasmic trafficking abnormalities in (G_4_C_2_)_149_ mice, our present study now provides insight on the interplay among SG biology, nucleocytoplasmic transport, G_4_C_2_/G_2_C_4_-associated pathologies and TDP-43 deposition.

## Methods

All animal procedures were performed in accordance with the National Institutes of Health Guide for Care and Use of Experimental Animals, and approved by the Mayo Clinic Institutional Animal Care and Use Committee (IACUC).

### Generation of (G_4_C_2_)_2_ and (G_4_C_2_)_149_ AAV vectors

AAV vectors were generated as previously described [9]. Briefly, the (G_4_C_2_)_2_ or (G_4_C_2_)_149_ repeats, along with 119 and 100 base pairs of the 5′ and 3′ flanking regions of the *C9orf72* gene, respectively, were inserted into an AAV expression vector (pAM/CBA-pl-WPRE-BGH) containing inverted terminal repeats of serotype 2. AAV-(G_4_C_2_)_2_ and AAV-(G_4_C_2_)_149_ particles were packaged into serotype 9 type capsid and purified using standard methods [64]. AAV was generated by co-transfection with helper plasmids into HEK293T cells. Cells were harvested 48 h after transfection, and lysed with 0.5% sodium deoxycholate and 50 Units/ml Benzonase (Sigma-Aldrich) by freeze thaw. The virus was then purified using a discontinuous iodixanol gradient, and the genomic titer of each virus determined by qPCR. AAV solutions were diluted with sterile phosphate-buffered saline (PBS).

### Neonatal viral injections

Intracerebroventricular injections of AAV were performed as previously described with some minor modifications [7, 28]. Briefly, a 32-gauge needle (product #7803–04, 0.5 in. custom length, point style 4, 12 degrees, Hamilton Company) attached to a 10 μl syringe (Hamilton Company) was inserted into the lateral ventricle of cryoanesthetized C57BL/6J pups at postnatal day 0. The needle was inserted at a 30-degree angle from the surface of the head, and held at a depth of approximately two millimeters. Two microliters (1.5E10 genomes/μl) of AAV2/9-(G_4_C_2_)_2_ or AAV2/9-(G_4_C_2_)_149_ solution was manually injected into each lateral ventricle. Following injection, pups were placed on a heated pad until they recovered from anesthesia, at which time they were placed back into their home cage.

### Behavioral tests

Cohorts of 3-month-old mice expressing (G_4_C_2_)_2_ (*n* = 17) or (G_4_C_2_)_149_ (n = 17), and 6-month-old mice expressing (G_4_C_2_)_2_ (*n* = 14) or (G_4_C_2_)_149_ (*n* = 11) underwent a battery of behavioral tests, including the open field assay, hanging wire test, and contextual fear conditioning test. All mice were acclimated to the testing room for 1–2 h prior to testing. All behavioral equipment was cleaned with 30% ethanol prior to use and between each animal. All mice were returned to their home cages and home room following completion of each test.

#### Open field assay

Mice were placed in the center of an open field apparatus, and allowed to explore the area for 15 min. Movement was monitored through the use of an overhead camera with AnyMaze software (Stoelting Co.), and total distance traveled was tracked.

#### Hanging wire test

A 2 mm thick wire tied to two vertical stands, approximately 55 cm apart, was maintained 35 cm above a layer of bedding material to prevent injury when an animal fell from the wire. Upon grasping the wire, the number of times the mouse fell from the wire within a 2 min time period were recorded.

#### Contextual fear conditioning test

This test was conducted in a test chamber with a grid floor capable of delivering an electric shock. Mice were initially placed in the chamber and left undisturbed for 2 min. An 80-dB white noise served as the conditioned stimulus (CS) and was presented for 30 s followed by a mild (2 s, 0.5 mA) foot shock serving as the unconditioned stimulus (US). A second CS-US pair was presented after 2 min, and the mouse was removed from the apparatus 30 s later and returned to its home cage. Twenty-four hours later, each mouse was returned to the test chamber and their freezing behavior was recorded for 5 min (in the absence of a CS-US pairing).

### RNA fluorescence in situ hybridization (FISH)

Mice were euthanized by carbon dioxide, and their brain and spinal cord were rapidly removed. Hemi-brains and the cervical half of the spinal cord was fixed in 4% paraformaldehyde for at least 48 h, and subsequently embedded in paraffin and sectioned at 5 μm (sagittal sections for brain, coronal sections for spinal cord). Paraffin sections were mounted on positively-charged glass slides, dried overnight, and the RNA FISH protocol was performed as previously described [9] with some modifications. In brief, tissue sections were deparaffinized in xylene, rehydrated through a series of ethanol solutions, permeabilized with ice cold 2% acetone/1xDEPC-PBS for five minutes, washed twice with DEPC-water, and then dehydrated through a series of ethanol solutions. To detect sense RNA foci, sections were incubated with pre-hybridization buffer (50% formamide [Midsci], 10% dextran sulfate [Millipore], 2x saline-sodium citrate buffer [SSC], 50 mM sodium phosphate buffer pH 7.0) for 20–30 min at 66 °C, and then hybridized for 24 h at 66 °C in a dark, humidified chamber with a fluorescently-labeled locked nucleic acid (LNA) probe [30] (TYE563-[CCCCGGCCCCGGCCCC]; Exiqon product number 500150, design id: 283117) diluted to a final concentration of 40 nM. To detect antisense foci, sections were first incubated with pre-hybridization buffer (50% formamide [MidSci], 10% dextran sulfate [Millipore], 2xSSC, 50 mM lithium phosphate buffer pH 7.0), and then incubated for 24 h at 60 °C in a dark, humidified chamber with an LNA probe [30] (TYE563-[GGGGCCGGGGCCGGGG]; Exiqon product number: 500150, design id: 345686) diluted to final concentration of 40 nM. Next, sections were washed with 2x SSC/0.1% Tween-20 at room temperature for 5 min, and then washed twice with pre-warmed 0.2x SSC at 60 °C for 10 min in the dark. Following these washes, slides were coverslipped using Vectashield mounting media with DAPI (Vector Laboratories). Representative images of sense and antisense RNA foci in the motor cortex, hippocampus, cerebellum and spinal cord were taken with an AxioImager Z1 fluorescent microscope (Carl Zeiss MicroImaging) under 63x magnification. Quantitative analysis of cells containing sense and antisense RNA foci in (G_4_C_2_)_149_ mice (*n* = 6 from each age group) was completed for the following regions: motor cortex (*n* = 300–400 cells), hippocampus (*n* = 500 cells), and cerebellar Purkinje cells (*n* = 100 cells).

### Immunohistochemistry and quantitative analysis of DPR protein inclusions

Tissue sections were deparaffinized in xylene and rehydrated through a series of ethanol solutions. Antigen retrieval was performed in distilled water or pH 9 Tris-EDTA (DAKO) for 30 min. Sections were then immunostained with rabbit polyclonal antibodies against poly(GA) (1:50,000), poly(GP) (1:10,000), poly(GR) (1:2500), poly(PA) (1:3500), or poly(PR) (1:500) using the DAKO Autostainer (Universal Staining System) and the DAKO+HRP system. Other antibodies used for immunohistochemical analysis were those for the detection of SG components (G3BP1 [1:100, Abclonal, A5342], eIF3Abclonal, A5342 [1:2000, Santa Cruz Biotechnology, sc-16,377], ataxin-2 [1:500, Proteintech, 21,776–1-AP]), RanGAP1 [1:100, Santa Cruz Biotechnology, sc-25,630], glial fibrillary acidic protein (GFAP, 1:2500, Biogenex) and neuronal nuclei (NeuN, 1:5000, Millipore). Sections were counterstained with hematoxylin, dehydrated through a series of ethanol and xylene washes, and coverslipped with Cytoseal mounting media (Thermo Fisher Scientific, Inc). Slides were scanned with a ScanScope® AT2 (Leica Biosystems) at 40x magnification, and representative images taken with ImageScope® software (v12.1; Leica Biosystems). Quantitative analysis of DPR protein burden in (G_4_C_2_)_149_ mice (*n* = 8 per age group) was performed by evaluating poly(GA), poly(GP), and poly(GR) protein inclusions in the motor cortex, and poly(PA) and poly(PR)-positive inclusions in the entire cortex. Quantitative analysis of the number of ataxin-2-positive inclusions in the motor cortex or hippocampus was performed on (G_4_C_2_)_149_-mice (*n* = 7 for 3 month; *n* = 9 for 12 month cohort). Quantification of the number of RanGAP1 nuclear invaginations in the cortex was performed on animals at 3 months [*n* = 6 for (G_4_C_2_)_2_-mice; n = 7 for (G_4_C_2_)_149_-mice] and 12 months of age [n = 6 for (G_4_C_2_)_2_-mice; n = 9 for (G_4_C_2_)_149_-mice].

### Immunofluorescence

Tissue sections were deparaffinized in xylene and rehydrated through a series of ethanol solutions. Antigen retrieval was performed by steaming in sodium citrate buffer (10 mM sodium citrate, 0.05% Tween-20, pH 6) for 30 min. Tissues were immunostained with antibodies against poly(GR) (1:1000, EMD Millipore, MABN778), TIA1 (1:50, Santa Cruz Biotechnology, sc-1751), ataxin-2 (1:500, Proteintech, 21,776–1-AP), and/or pTDP-43 (1:1000, Cosmo, CAC-TIP-PTD-P02) overnight at 4 °C. Sections were subsequently incubated with corresponding secondary antibodies (1:500, Molecular Probes) and coverslipped with Vectashield with DAPI. Images were taken with Zeiss LSM 800 confocal microscope.

### Digital pathology

Percentage of GFAP immunoreactivity and NeuN-positive neuronal density were quantified using Aperio® ePathology technology (Leica Biosystems) as previously described [9]. Briefly, stained slides were scanned and digitized with ScanScope® AT2 (Leica Biosystems), and ImageScope® software (v12.1; Leica Biosystems) was used to annotate the cortex on serial sections stained for GFAP or NeuN. GFAP immunolabeling was quantified by a custom-designed positive pixel count algorithm [45], which provided the percent burden of positively-stained pixels per each annotated region as the output parameter (*n* = 8 per age group). NeuN-positive neurons were quantified using an algorithm designed to detect nuclei. The output parameter was the number of NeuN-positive neurons per mm^2^ of the annotated cortical region (*n* = 6 per age group).

### pTDP-43 immunohistochemistry

Immunohistochemistry for pTDP-43 was performed using the VECTASTAIN® Elite_®_ ABC (Vector Laboratories) kit, with minor modifications to the manufacturer’s protocol. Tissue sections were deparaffinized and rehydrated in a graded series of xylene and ethanol washes prior to antigen retrieval by steaming for 30 min in sodium citrate buffer (10 mM sodium citrate, 0.05% Tween-20, pH 6.0). Slides were allowed to slowly cool for 15–20 min, and then gently flushed with distilled water for 15 min. Tissue sections were incubated with Dako Dual Endogenous Enzyme Block (DAKO) and then rinsed three times with 1x PBS for five minutes at room temperature. Sections were blocked with 2% normal goat serum in 1xPBS for one hour at room temperature, and then incubated with pTDP-43 antibody (pS409/410, generated in-house [9, 10], 1:500) overnight at 4 °C. The following day, slides were washed with 1xPBS at room temperature (three 5 min washes), and then incubated with biotinylated goat anti-rabbit secondary (1:200) for 2 h at room temperature. Slides were subsequently washed with another round of 1xPBS (three 5 min washes), incubated with avidin-biotin complex solution for 30 min, and washed again in 1xPBS (three 10 min washes). 3,3′-diaminobenzidine (Acros Organics) was activated with hydrogen peroxide, and the reaction stopped by rinsing slides in distilled water. Slides were then counterstained with hematoxylin, dehydrated through a series of ethanol washes and xylene, and coverslipped with Cytoseal mounting media (Thermo Fisher Scientific, Inc). Slides were scanned with a ScanScope® AT2 (Leica Biosystems) at 40x magnification. Quantitative analysis of pTDP-43 inclusions in the cortex of (G_4_C_2_)_149_ (*n* = 6 per age group) was performed with ImageScope® software (v12.1; Leica Biosystems).

### Statistical analysis for behavioral studies and quantification of pathology

To determine whether statistically significant differences in RNA foci, DPR protein burden or pTDP-43 inclusions were observed among (G_4_C_2_)_149_ mice of different ages, 1-way ANOVA followed by a Tukey’s posthoc test for multiple comparisons was used. To compare NeuN and GFAP immunoreactivity between (G_4_C_2_)_2_ and (G_4_C_2_)_149_ mice at 3 or 6 months of age, a 2-way ANOVA with a Tukey’s posthoc test was used. To compare the performance of (G_4_C_2_)_2_ and (G_4_C_2_)_149_ mice in behavioral tests (i.e., the open field assay, the hanging wire test and the contextual fear conditioning test), unpaired two-tailed *t* tests were performed. All statistical analyses were performed in GraphPad Prism. Data are presented as mean +/− SEM, with *p* < 0.05 considered statistically significant.

## Results

### Behavioral abnormalities coincide with neurodegeneration in (G_4_C_2_)_149_ mice

**Fig. 1:**
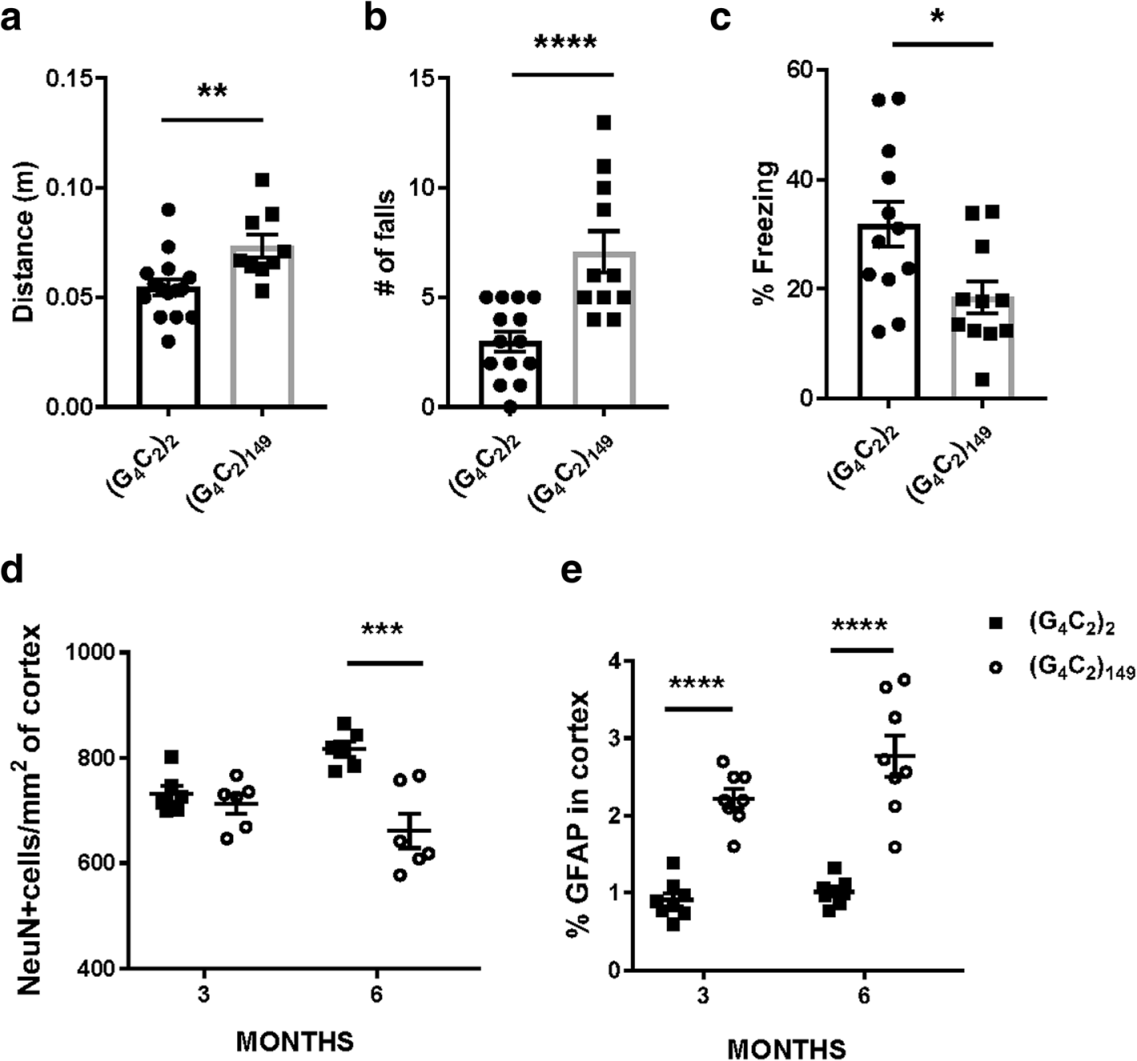
(G_4_C_2_)_149_–mice exhibit hyperactivity, motor deficits, and memory loss at 6 months of age. **a** Significant increase in distance traveled in (G_4_C_2_)_149_ mice in open field analysis indicative of hyperactivity. **b** Hanging wire test revealed an increase in the number of falls in (G_4_C_2_)_149_ mice relative to age-matched (G_4_C_2_)_2_ mice. **c** Cognitive function was assessed by contextual fear conditioning, which demonstrated a significant reduction in freezing in (G_4_C_2_)_149_ mice, indicative of cognitive impairment. **d** Quantitative analysis of the number of NeuN-positive neurons per mm^2^ of cortex reveals neuronal loss in (G_4_C_2_)_149_ mice at 6 months of age, but no difference with (G_4_C_2_)_2_ mice at 3 months. **e** The percent of GFAP immunoreactivity in the cortex demonstrates a significant increase in gliosis in (G_4_C_2_)_149_ mice at both 3 and 6 months relative to control (G_4_C_2_)_2_ mice. Data represent the mean ± SEM. *p < 0.05, **p < 0.01,***p < 0.001, and ****p < 0.0001 as analyzed by unpaired two-tailed *t* tests (**a**-**c**) or 2-way ANOVA (**d-e**) followed by Tukey’s post-hoc analysis

Intracerebroventricular injections were performed to deliver AAV encoding (G_4_C_2_)_2_ or the expanded (G_4_C_2_)_149_ to the murine CNS on postnatal day 0, followed by behavioral and histopathological analysis of c9FTD/ALS features. To first characterize behavioral consequences of CNS (G_4_C_2_)_149_ expression, we subjected mice to an open field assay, a hanging wire test, and a contextual fear conditioning test. Consistent with our (G_4_C_2_)_66_ mouse model [9], 3- and 6-month-old (G_4_C_2_)_149_ mice displayed hyperactivity in the open field assay (Fig. 1a, Additional file 1: Figure S1a). However, motor deficits, as evidenced by an increased number of falls in the hanging wire test, were observed in 6- but not 3-month-old (G_4_C_2_)_149_ mice (Fig. 1b, Additional file 1: Figure S1b). (G_4_C_2_)_149_ mice also exhibited signs of cognitive dysfunction in the contextual fear conditioning test at 6 months of age. Specifically, the significant decrease in the amount of time (G_4_C_2_)_149_ mice spent freezing in this test is indicative of their impaired ability to associate the test environment with a mild electrical shock, and is suggestive of hippocampal dysfunction (Fig. 1c, Additional file 1: Figure S1c).

To determine whether behavioral abnormalities are associated with markers of neurodegeneration in (G_4_C_2_)_149_ mice, we evaluated astrogliosis and neuronal loss. Compared to (G_4_C_2_)_2_ mice, (G_4_C_2_)_149_ mice showed significantly elevated immunoreactivity for the astrocytic marker GFAP in the cortex at both 3 and 6 months of age (Fig. 1e**,** Additional file 1: Figure S2a). However, a significant loss of NeuN-positive cortical neurons was only evident at 6 months of age, but not at 3 months of age (Fig. 1d, Additional file 1: Figure S2b), suggesting that astrogliosis precedes neurodegeneration in (G_4_C_2_)_149_ mice. Collectively, our findings indicate that the early development of astrogliosis and hyperactivity precede significant neurodegeneration in (G_4_C_2_)_149_ mice, and that the development of cognitive and motor deficits follow the time course of neuronal loss (Fig. 1, Additional file 1: Figure S1–2).

### (G_4_C_2_)_149_ mice develop sense and antisense RNA foci

**Fig. 2:**
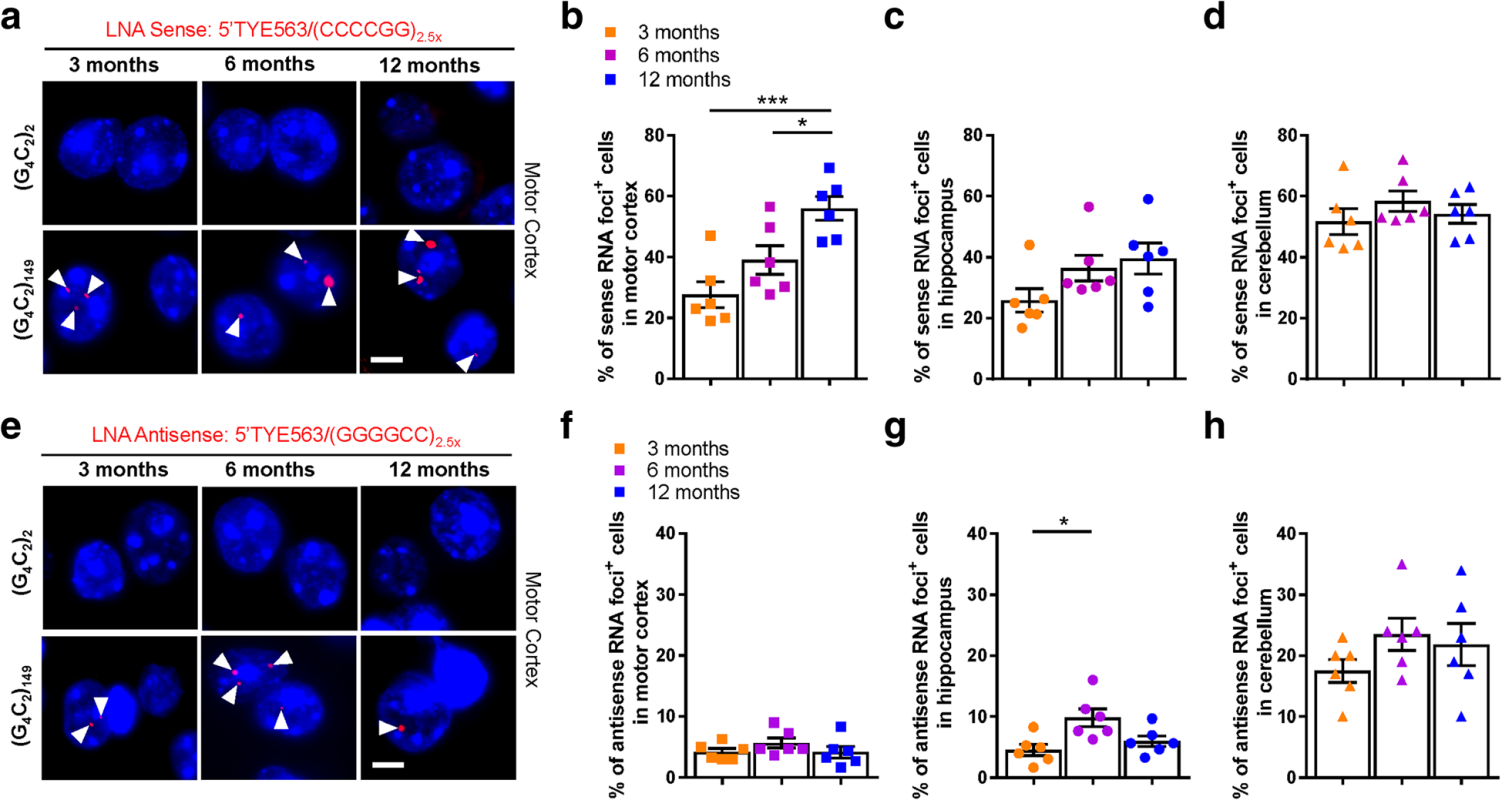
Sense and antisense RNA foci observed in the CNS of (G_4_C_2_)_149_–mice. **a**) Sense RNA foci (white arrowheads) were detected throughout the CNS with representative images from the motor cortex of 3, 6, and 12 month (G_4_C_2_)_149_–mice (bottom panel, **a**), but not in control (G_4_C_2_)_2_–mice (top panel, **a**). **b-d** Percentage of cells containing sense RNA foci in the motor cortex (**b**), CA1 through CA3 region of the hippocampus (**c**), and Purkinje layer of the cerebellum (**d**) in (G_4_C_2_)_149_ mice at 3, 6, and 12 months of age. **e**) Antisense RNA foci (white arrowheads) were detected throughout the CNS in (G_4_C_2_)_149_–mice (bottom panel, **e**) but not in control (G_4_C_2_)_2_–mice (top panel, **e**) at 3, 6, and 12 months of age, with representative images from the motor cortex. **f-h**) Percentage of cells containing antisense RNA foci in the motor cortex (**f**), CA1 through CA3 region of the hippocampus (**g**), and Purkinje layer of the cerebellum (**h**) in 3, 6, and 12-month (G_4_C_2_)_149_–mice. Data represent the mean ± SEM. *P < 0.05 and ***P < 0.001, as analyzed by one-way ANOVA followed by Tukey’s post-hoc analysis. Scale bar represents 5 μm

To determine whether behavioral abnormalities and neurodegeneration in (G_4_C_2_)_149_ mice are related to the accumulation of *C9orf72* repeat-associated pathologies, we first performed RNA FISH using a probe to detect sense G_4_C_2_ repeat RNA. This analysis revealed intranuclear foci of G_4_C_2_ repeat RNA (sense foci) in various regions of the CNS in (G_4_C_2_)_149_ mice, including the motor cortex (Fig. 2a, bottom panel), the hippocampus, the Purkinje cell layer of the cerebellum, the thalamus, and, to a lesser extent, the ventral horn of the spinal cord (Additional file 1: Figure S3a). No sense RNA foci were detected in (G_4_C_2_)_2_ mice at any age (Fig. 2a, top panel).

To assess whether aging exacerbates RNA foci accumulation, we quantified sense RNA foci in (G_4_C_2_)_149_ mice at 3, 6, and 12 months of age in brain regions with the highest foci burden (i.e., motor cortex, hippocampal CA1–CA3 regions, cerebellar Purkinje cell layer). The number of foci-positive cells was approximately 28, 39, and 56% in the motor cortex of (G_4_C_2_)_149_ mice at 3, 6, and 12 months of age, respectively, with a significant increase in foci burden detected in 12-month-old mice compared to younger mice (Fig. 2b). Of these foci-positive-cells, approximately 70% harbored one or two RNA foci per cell, while the remaining 30% of cells contained three or more foci (with no significant difference across age groups; Additional file 1: Figure S3d). In the hippocampus, approximately 26 to 40% of cells in the CA1–CA3 regions contained RNA foci in (G_4_C_2_)_149_ mice but no significant difference was seen among the three age groups (Fig. 2c). Consistent with results from c9FTD/ALS postmortem tissues [13], the cerebellar Purkinje cell layer had the highest burden of sense RNA foci among the three regions, with approximately 52 to 58% of cells containing nuclear RNA foci (Fig. 2d). However, as in the hippocampus, the percentage of sense foci in Purkinje cells did not significantly differ with age.

Given that foci of antisense G_2_C_4_ transcripts are also detected in postmortem brain tissues of c9FTD/ALS patients [12, 13, 18], and that inverted terminal repeats present within the AAV vector both 5′ and 3′ of the G_4_C_2_ repeat have been shown to exert promoter-like activity [16], we next evaluated whether antisense G_2_C_4_ repeat RNA was present in (G_4_C_2_)_149_ mice by RNA FISH. Antisense RNA foci were observed in the same brains regions in which sense RNA foci were detected, albeit at a much lower frequency (Fig. 2e bottom panel, Additional file 1: Figure S3b). Approximately 4 to 6% of cells in the motor cortex, and 5 to 10% of cells in the hippocampus contained antisense RNA foci with minimal differences in frequency seen across the three age groups (Fig. 2f-g, Additional file 1: Figure S3d). Again, the cerebellar Purkinje cell layer exhibited the highest burden of foci among the three assessed regions, with approximately 18 to 24% of cells bearing antisense foci among the three age groups (Fig. 2h). Overall, age appeared to have minimal effect on antisense RNA foci burden.

### (G_4_C_2_)_149_ mice develop sense and antisense DPR protein pathology

**Fig. 3:**
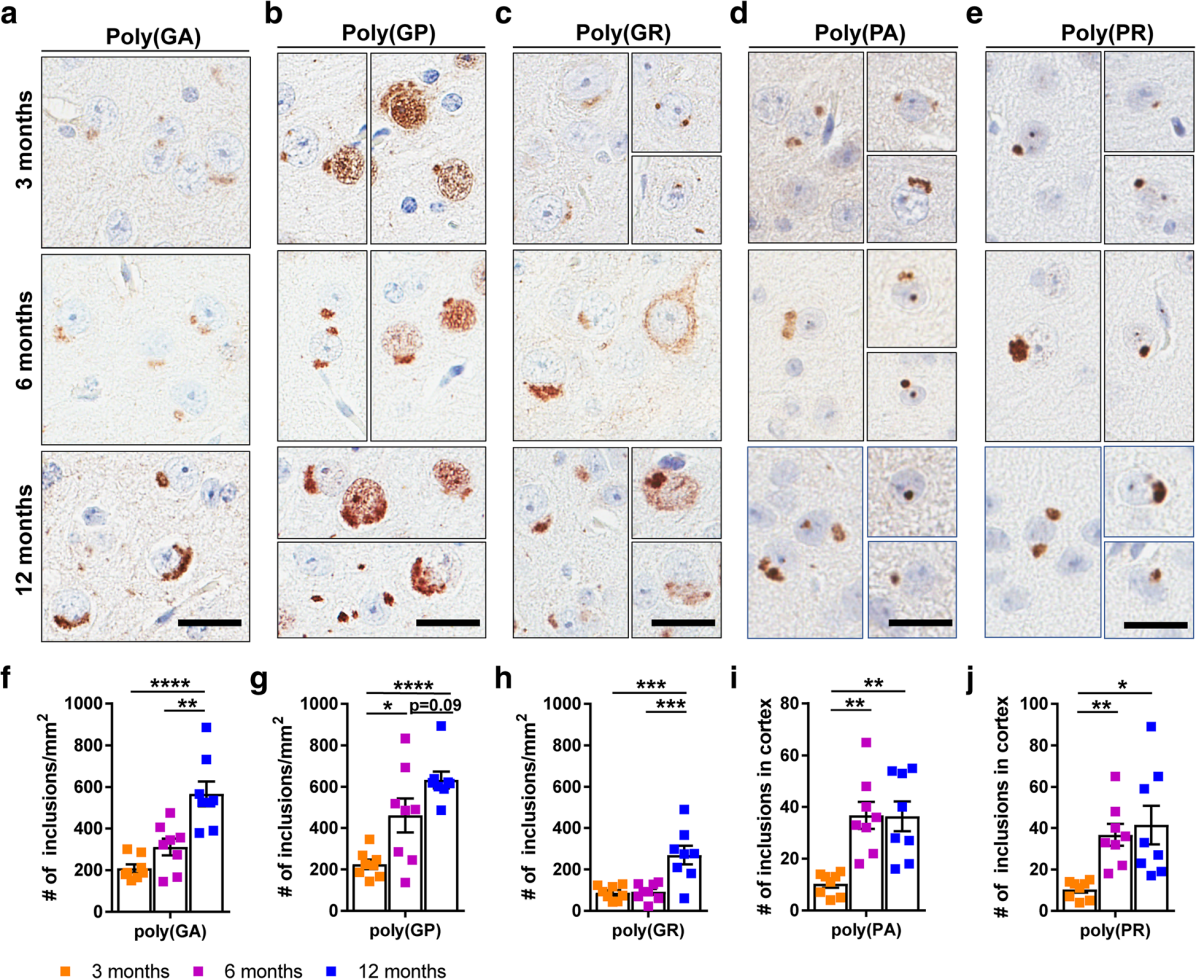
Sense and antisense DPR protein inclusions in (G_4_C_2_)_149_–mice. **a-e** Representative images of immunohistochemical analysis of poly(GA), poly(GP), poly(GR), poly(PA), and poly(PR) proteins in the motor cortex of (G_4_C_2_)_149_ mice at 3, 6, and 12 months of age. **f-h** Quantitative analysis of the number of poly(GA) (**f**), poly(GP) (**g**), or poly(GR) (**h**) inclusions observed per mm^2^ of motor cortex in (G_4_C_2_)_149_–mice at 3, 6, and 12-months. **i-j** Quantitative analysis of poly(PA) (**i**) or poly(PR) (**j**) inclusions observed in the cortex of 3, 6, and 12-month (G_4_C_2_)_149_–mice. Data represent the mean ± SEM. *p < 0.05, **p < 0.01,***p < 0.001, and ****p < 0.0001 as analyzed by 1-way ANOVA followed by Tukey’s post-hoc analysis. Scale bar represents 20 μm

Neuronal inclusions of DPR proteins generated from sense and antisense repeat RNA are a prominent hallmark of c9FTD/ALS. To characterize DPR protein pathology in the CNS of (G_4_C_2_)_2_ and (G_4_C_2_)_149_ mice, a panel of polyclonal antibodies specific to each of the DPR proteins was used for immunohistochemical analyses. We observed cytoplasmic aggregates immunopositive for poly(GA), poly(GP), or poly(GR) in various neuroanatomical regions of (G_4_C_2_)_149_ mice that were reminiscent of inclusions detected in c9FTD/ALS postmortem brains (Fig. 3). From 3 months of age, these inclusions were present in all layers of the cortex, CA1–CA3 regions of the hippocampus (less frequently observed in the dentate gyrus), the cerebellum, and the spinal cord (Additional file 1: Figure S4a-c). Additionally, diffuse nuclear poly(GP) was readily detected (Fig. 3b, Additional file 1: Figure S4a-c), as was diffuse cytosolic poly(GR) (Fig. 3c, Additional file 1: Figure S4a-c). Poly(GA), poly(GP) and poly(GR) inclusions increased in size, number, and stained more intensely across aging, suggestive of progressive aggregation over time. Indeed, quantitative analysis of poly(GA) (Fig. 3f), poly(GP) (Fig. 3g), and poly(GR) (Fig. 3h) inclusions in the motor cortex demonstrated an age-dependent increase from 3 to 12 months of age. Poly(GA) and poly(GP) inclusions were more frequent than poly(GR) inclusions (Fig. 3f-h), recapitulating the inclusion profile in c9FTD/ALS [35].

Of importance, we also detected inclusions throughout the cortex that were immunopositive for poly(PA) (Fig. 3d) and poly(PR) (Fig. 3e), the two DPR proteins uniquely produced from antisense G_2_C_4_ RNA. However, their frequency was much lower relative to poly(GA), poly(GP) and poly(GR) inclusions, again mimicking observations from c9FTD/ALS postmortem brain tissues [18, 35, 65]. The number of poly(PA) and poly(PR) inclusions increased with age, with the majority of inclusions detected in the cortex and exhibiting a cytoplasmic localization, although a small fraction did localize to the nucleus (Fig. 3d-e, i-j). Poly(PA) and poly(PR) inclusions were also occasionally observed in the hippocampus of (G_4_C_2_)_149_ mice at 12 months of age, but were rarely found in other regions of the CNS (not shown). To assess whether repeat length influences antisense DPR protein production, we examined mice injected with an AAV vector encoding a shorter 66-mer G_4_C_2_ repeat [9]. (G_4_C_2_)_66_ mice were positive for both poly(PA) and poly(PR) inclusions at 6 months of age; however, the frequency of these inclusions was significantly lower relative to those in mice expressing 149 G_4_C_2_ repeats (Additional file 1: Figure S5). Neither sense nor antisense DPR protein pathology was detected in the CNS of (G_4_C_2_)_2_ mice at any age (Additional file 1: Figure S6). These results indicate that the expression of (G_4_C_2_)_149_ in mice is associated with an age-dependent accumulation of DPR proteins derived from both sense and antisense repeat RNA, providing a valuable model to test the efficacy of therapeutic strategies targeting either sense (G_4_C_2_) or antisense (G_2_C_4_) RNA transcripts or DPR proteins.

### (G_4_C_2_)_149_ mice develop progressive pTDP-43 pathology

**Fig. 4:**
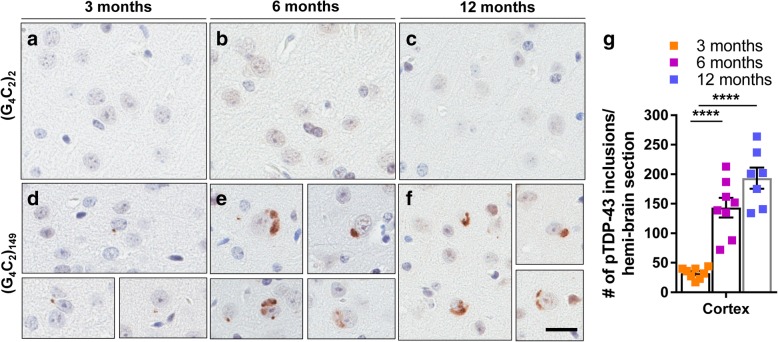
Cortical pTDP-43 (pS409/410) pathology detected in (G_4_C_2_)_149_–mice. **a-f** Representative images of immunohistochemical analysis of pTDP-43 in the cortex of (G_4_C_2_)_2_ (**a-c**) and (G_4_C_2_)_149_ mice (**d-f**) at 3, 6, and 12 months of age. **g** Quantitative analysis of pTDP-43 inclusions in the cortex. Data represent the mean ± SEM. ****p < 0.0001 as analyzed by 1-way ANOVA followed by Tukey’s post-hoc analysis. Scale bar represents 20 μm

Since pTDP-43 pathology tracks with neurodegeneration in c9FTD/ALS [34], we evaluated the temporal profile of pTDP-43 pathology in (G_4_C_2_)_149_-AAV mice. Inclusions containing pTDP-43 were absent in (G_4_C_2_)_2_ mice (Fig. 4a-c, Additional file 1: Figure S7a), but small cytoplasmic pTDP-43 inclusions reminiscent of those observed in c9FTD/ALS were detected in the cortex (Fig. 4d) and hippocampus (Additional file 1: Figure S7b top panel) of 3-month-old (G_4_C_2_)_149_ mice. By 6 and 12 months of age, the number of cytoplasmic pTDP-43 inclusions was significantly increased in the cortex (Fig. 4e-g) and the hippocampus (Additional file 1: Figure S7b middle, bottom panel). These findings indicate that aging exacerbates the accumulation and aggregation of pTDP-43 in (G_4_C_2_)_149_ mice.

### (G_4_C_2_)_149_ mice exhibit aggregation of stress granule-associated proteins and nucleocytoplasmic transport defects

**Fig. 5:**
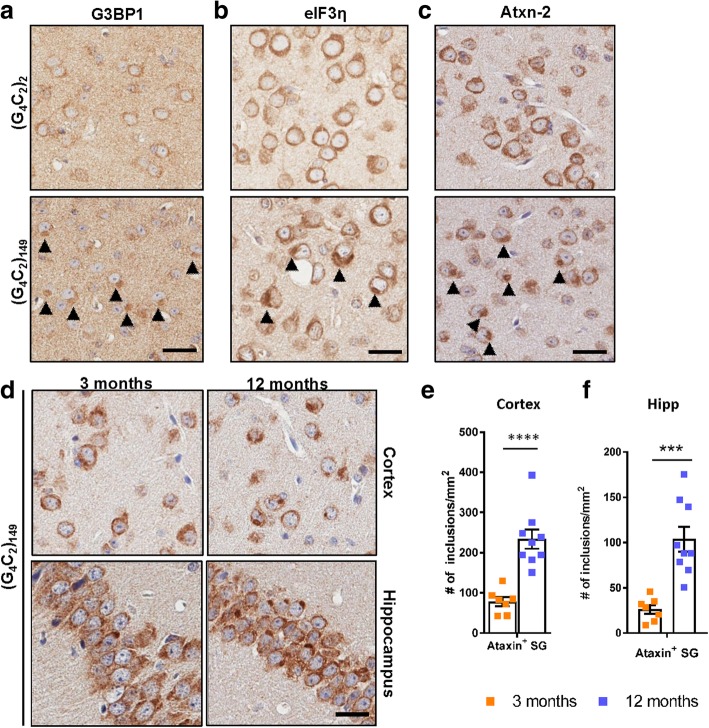
Deposition of stress granule-associated proteins in (G_4_C_2_)_149_–mice. **a-c** Representative images of immunohistochemical analysis of G3BP1 (**a**), eIF3η (**b**), and ataxin-2 (**c**) in the cortex of (G_4_C_2_)_2_ and (G_4_C_2_)_149_ mice at 12 months of age, with inclusions indicated by black arrowheads. **d** Representative images of ataxin-2-positive inclusions in the cortex and hippocampus of (G_4_C_2_)_149_ mice at either 3 or 12 months of age. **e-f** Quantitative analysis of the number of ataxin-2 inclusions per mm^2^ of cortex (**e**) or hippocampus (**f**) in (G_4_C_2_)_149_ mice. Data represent the mean ± SEM. ****p < 0.0001 as analyzed by unpaired two-tailed *t* test. Scale bar represents 20 μm

**Fig. 6:**
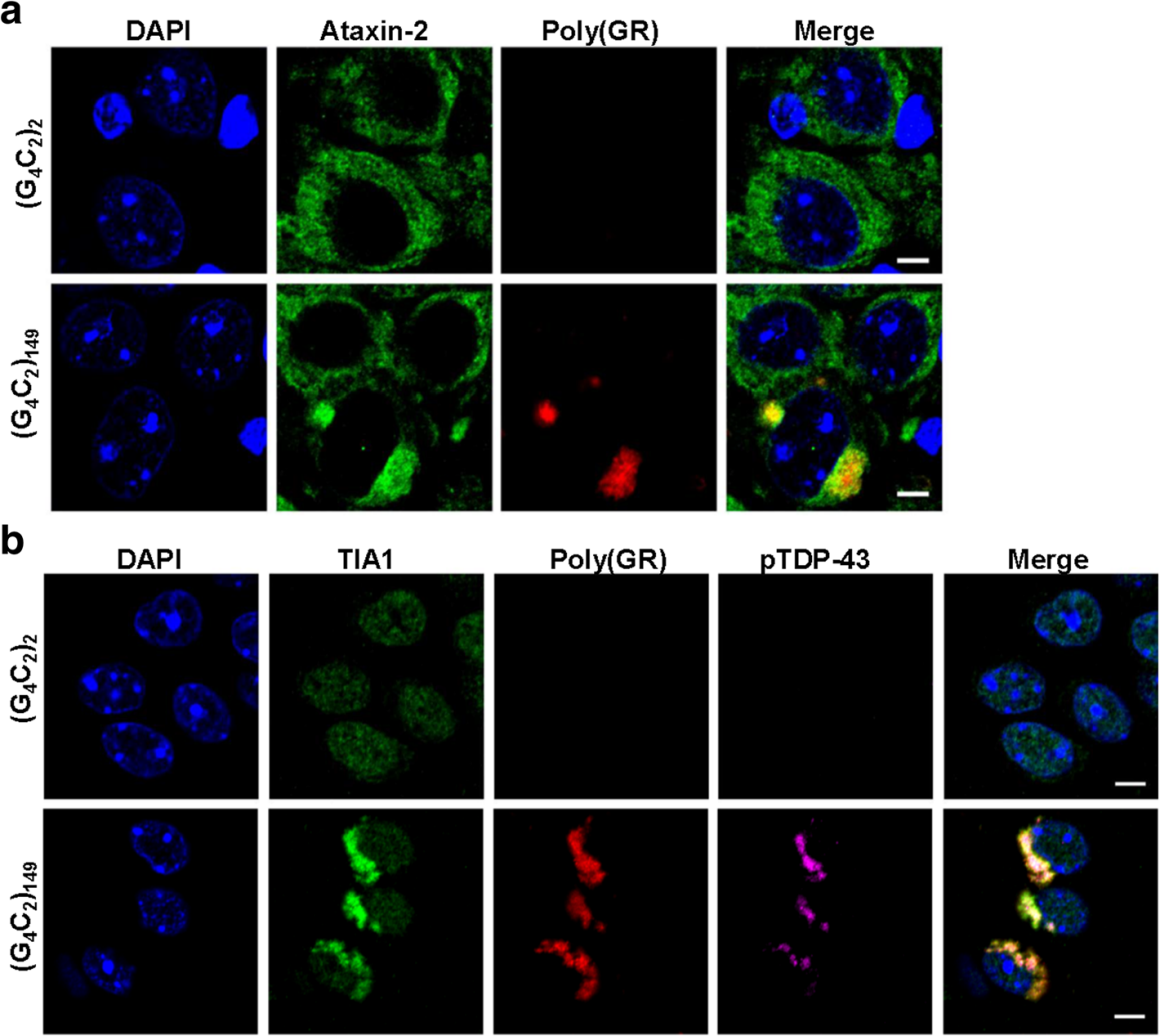
Stress granule markers colocalize with poly(GR) and pTDP-43 in (G_4_C_2_)_149_–mice. **a-b** Representative immunofluorescent images depicting colocalization between poly(GR) and either ataxin-2 (**a**) or TIA-1 and pTDP-43 (**b**) in (G_4_C_2_)_149_ mice, with no pathology detected in control (G_4_C_2_)_2_ mice. Nuclei are labeled with DAPI. Scale bar represents 5 μm

Given that TDP-43 localizes to SGs [32, 38], and that aberrant SG responses have been implicated in the pathogenesis of c9FTD/ALS [6, 8, 20], we investigated whether the expression of (G_4_C_2_)_149_ in the murine CNS was associated with the abnormal deposition of SG-resident proteins. To do so, we immunostained mouse brain sections for essential protein components and modulators of SG assembly, including: G3BP stress granule assembly factor 1 (G3BP1), ataxin-2, and eukaryotic initiation-factor 3η (eIF3η). Inclusions immunopositive for these three SG proteins were detected in the brains of (G_4_C_2_)_149_ mice but not control (G_4_C_2_)_2_ mice (Fig. 5a-c, Additional file 1: Figure S8a). Of note, the number of ataxin-2-positive inclusions increased in an age-dependent manner in the cortex and hippocampus (Fig. 5d-f), suggesting that the aberrant deposition of SG components, like that of pTDP-43, was both chronic and progressive. Since we recently reported that another SG component, T cell intracellular antigen 1 (TIA-1), colocalizes to poly(GR)-positive inclusions in the brains of (G_4_C_2_)_66_ and (G_4_C_2_)_149_ mice [61], we examined whether the same was true for ataxin-2. As shown in Fig. 6a-b, both ataxin-2 and TIA1 colocalized with aggregated, but not diffuse, poly(GR) in (G_4_C_2_)_149_ mice. What is more, TIA1/poly(GR)-positive inclusions were also immunopositive for pTDP-43 (Fig. 6b, Additional file 1: Figure S8b).

**Fig. 7:**
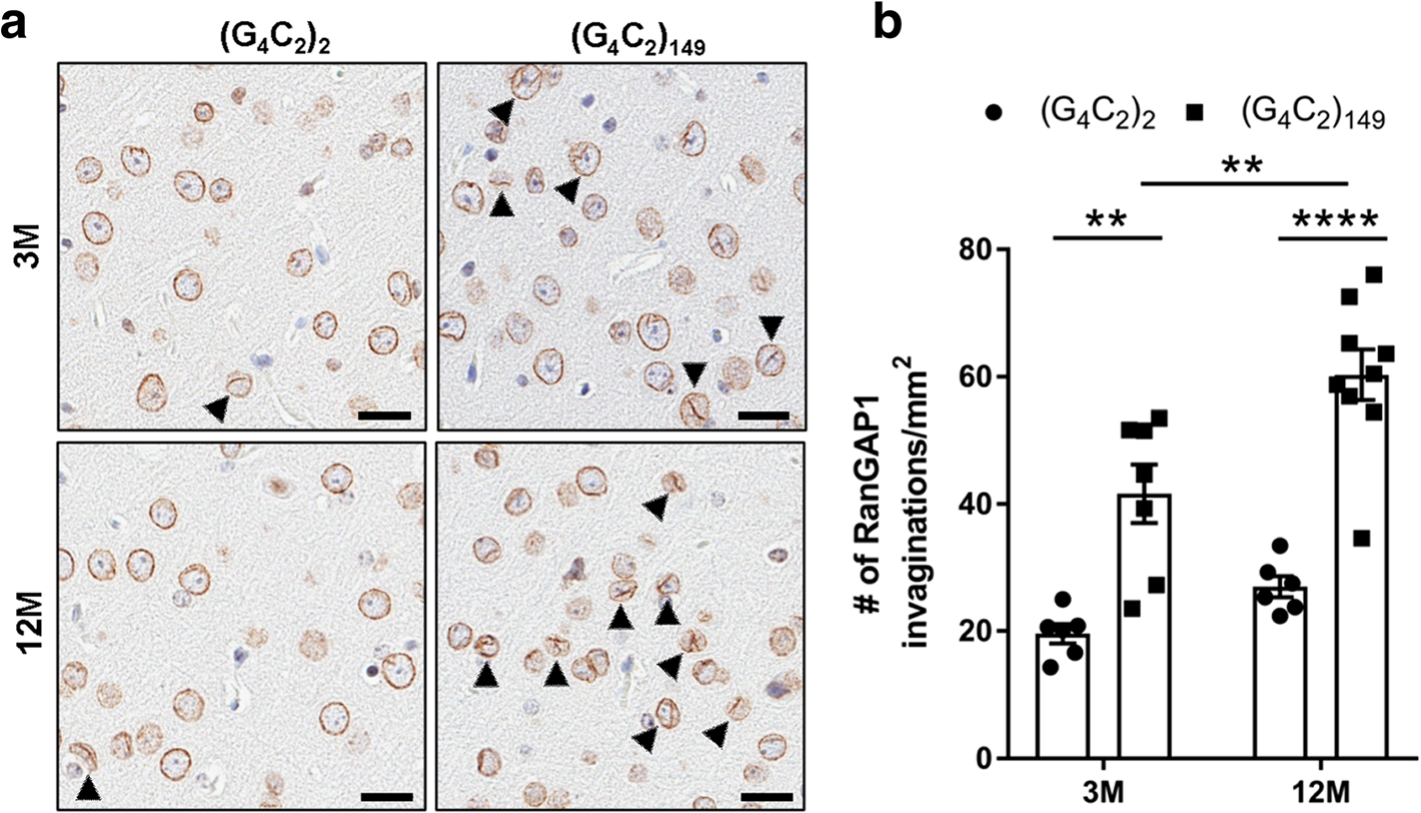
Mislocalization of RanGAP1 in (G_4_C_2_)_149_–mice. **a** Representative images of immunohistochemical analysis of RanGAP1 in the cortex of (G_4_C_2_)_2_ and (G_4_C_2_)_149_ mice at 3 and 12 months of age, with nuclear invaginations indicated by black arrowheads. **b** Quantitative analysis of the number of RanGAP1-positive nuclear invaginations per mm^2^ of cortex. Data represent the mean ± SEM. **p < 0.01, ****p < 0.0001 as analyzed by 2-way ANOVA followed by Tukey’s post-hoc analysis. Scale bar represents 20 μm

Considering the recent finding that aberrant SG assembly can disrupt nucleocytoplasmic transport [59], we examined the cellular distribution of RanGAP1, an essential regulator of nucleocytoplasmic transport that has been shown to be mislocalized in *Drosophila* expressing the G_4_C_2_ repeat, as well as in iPSCs and motor cortex from a *C9orf72* ALS patient [60]. In (G_4_C_2_)_149_ mice, we observed a significant increase in the abnormal localization of RanGAP1 to nuclear invaginations relative to control (G_4_C_2_)_2_ mice as early as 3 months of age (Fig. 7). Collectively, these results provide new insight into the mechanism by which pTDP-43 accumulates in (G_4_C_2_)_149_ mice, and suggest that aberrant SG dynamics, poly(GR) deposition and nucleocytoplasmic transport defects are key drivers of TDP-43 pathology in c9FTD/ALS.

## Discussion

We developed a novel mouse model that accurately recapitulates several pathologic features of c9FTD/ALS, including both sense and antisense RNA foci and DPR protein burden. These features are accompanied by gliosis, neuronal loss, pTDP-43 aggregation, as well as motor and cognitive dysfunction. (G_4_C_2_)_149_-mice also display nucleocytoplasmic transport defects and an abnormal accumulation of SG-resident proteins within inclusions immunopositive for poly(GR) and pTDP-43, findings that provide new insight into the relationship between *C9orf72* repeat-associated pathologies and TDP-43 proteinopathy. Given that aberrant SG assembly has been shown to perturb nucleocytoplasmic trafficking [59], and SG-resident proteins have been detected within TDP-43 inclusions [5, 26, 32, 40], these results combined with the current data suggest SG dynamics, nucleocytoplasmic transport, and TDP-43 are key hubs in a pathogenic feedforward cycle.

To elucidate the role of poly(GR) in these events, in a prior study we used alternate codons to drive expression of poly(GR)_100_ in the murine CNS, in which virtually no pTDP-43 pathology or nucleocytoplasmic transport defects were detected [61]. Furthermore, the poly(GR) in these GFP-(GR)_100_-expressing mice, which was diffuse and cytoplasmic, did not colocalize with SG proteins. While colocalization with other DPRs has yet to be assessed, these data suggest that only aggregated poly(GR) (as occurs in (G_4_C_2_)_149_-mice) induces spontaneous SG formation or sequesters SG-resident proteins, enhances TDP-43 pathology, and perturbs nucleocytoplasmic transport. The data also suggest that merely expressing poly(GR) in vivo is not sufficient to cause its aggregation. Rather, in order to aggregate, poly(GR) may need to reach a certain threshold level and/or be accompanied by additional *C9orf72* repeat-associated pathologies, such as poly(GA) [58]. Since G_4_C_2_-repeat RNA, like poly(GR), stimulates SG formation, sequesters ribosomal subunits, and inhibits global translation [15, 20, 53, 61], a synergistic effect of G_4_C_2_-repeat RNA and poly(GR) may promote TDP-43 phosphorylation and aggregation through translational inhibition and chronic SG formation.

Findings from bacterial artificial chromosome (BAC) transgenic mouse models, which express expanded *C9orf72* repeats at lower levels than in our (G_4_C_2_)_149_-mice and (G_4_C_2_)_66_-mice (reviewed [3]), also suggest that repeat RNA and/or DPR protein concentrations influence the development of key c9FTD/ALS features. Sense and antisense RNA foci and certain DPR proteins were observed in the four BAC mouse models (reviewed [3]). However, two models showed no abnormalities in TDP-43 [48, 49]; one developed an increase in insoluble pTDP-43 in brain lysates but displayed no TDP-43 mislocalization or aggregation [24], and the model with the most aggressive phenotype exhibited TDP-43 aggregates at end-stage disease [33]. Of the two models that showed TDP-43 abnormalities (i.e., increased TDP-43 phosphorylation in the model created by Lagier-Tourenne and colleagues [24], and TDP-43 pathology in the mice from the Ranum group [33]), poly(GR) was examined only in the former and found to form inclusions. While this may support our finding that poly(GR) in the presence of G_4_C_2_ repeat RNA is linked to TDP-43 abnormalities, a more comprehensive characterization of all DPR proteins, especially poly(GR), in these models is required to probe this question more thoroughly. Nevertheless, data from human postmortem tissues offer support linking poly(GR) to TDP-43 pathology. Saberi and colleagues have reported that poly(GR) inclusions co-localize with TDP-43 in dendritic structures in the motor cortex of patients with c9ALS [50]. In addition, our recent findings demonstrate that the density and distribution of poly(GR) inclusions in *C9orf72* repeat expansion carriers differs from that of poly(GA) and poly(GP), with poly(GR) inclusions associating with neurodegeneration [51] similarly to what has been reported for TDP-43 pathology [34]. Moreover, the relative abundance of poly(GR) inclusions is highest in patients with the most severe clinicopathologic phenotype (frontotemporal lobar degeneration with motor neuron disease) [51]. These data, along with those from the present study and from our poly(GR) mouse model [61], offer compelling evidence for a role of poly(GR) in eliciting TDP-43 pathology and neurodegeneration. This contrasts with early studies investigating associations between the neuroanatomical distribution of DPR proteins and neurodegeneration in *C9orf72* expansion carriers [34], which largely focused on poly(GA) and poly(GP). Indeed, the contribution of DPR proteins versus repeat RNA to c9FTD/ALS pathogenesis has been, and remains, a topic of vigorous debate [1]. However, our production of a mouse model with both sense and antisense RNA and DPR protein pathology will help us answer this important question by dramatically facilitating both mechanistic and preclinical studies.

## Conclusion

Our in vivo model of c9FTD/ALS, the first to robustly recapitulate hallmark features derived from both sense and antisense *C9orf72* repeat-associated transcripts, and to display neurodegeneration and behavioral impairments, provides novel insight into the mechanism driving TDP-43 proteinopathy in c9FTD/ALS. In future studies, it will be of particular interest to evaluate the relationship(s) between the primary *C9orf72* repeat-associated pathologies, the abnormal deposition of SG-resident proteins, nucleocytoplasmic trafficking defects, and TDP-43 pathology, and to determine the critical pathologic features that need to be targeted in order to rescue neuronal loss and behavioral deficits. Such studies could be accomplished through the use of antisense oligonucleotides (ASOs) that target sense versus antisense repeats, or antibodies that target specific DPR proteins. Moreover, given the abundance of SG protein pathology in our (G_4_C_2_)_149_-mice, the model provides the means to test potential therapeutics targeting the SG pathway in vivo, such as ataxin-2 ASOs [4] or small molecules (i.e., ISRIB) [59]. These approaches, used in parallel or simultaneously, will also provide new insight into the feasibility and/or requirements of reversing pTDP-43 aggregation, neurodegeneration and functional deficits.

## Additional file

Additional file 1: Supplementary Data. (PDF 42745 kb)

## Abbreviations

AAV: Adeno-associated virus
ALS: Amyotrophic lateral sclerosis
ASO: Antisense oligonucleotide
BAC: Bacterial artificial chromosome
C9orf72: Chromosome 9 open reading frame 72
CNS: Central nervous system
CS: Conditioned stimulus
DPR: Dipeptide repeat
eIF3η: Eukaryotic initiation factor 3η
FISH: fluorescence in situ hybridization
FTD: Frontotemporal dementia
G3BP1: G3BP stress granule assembly factor 1
GA: Glycine-alanine
GFAP: Glial fibrillary acidic protein
GP: Glycine-proline
GR: Glycine-arginine
PA: Proline-alanine
PR: Proline-arginine
RAN: Repeat-associated non-ATG
SG: Stress granules
TDP-43: TAR DNA-binding protein 43
TIA-1: T cell intracellular antigen 1
US: Unconditioned stimulus
