# Supplementary material for: Aberrant deposition of stress granule-resident proteins linked to C9orf72-associated TDP-43 proteinopathy
Source: Mol Neurodegener. 2019 Feb 15;14:9. doi: 10.1186/s13024-019-0310-z (PMC6377782; doi:10.1186/s13024-019-0310-z)
Supplement: Supplementary file 1 — Supplementary Data. (PDF 42745 kb) [file 13024_2019_310_MOESM1_ESM.pdf]

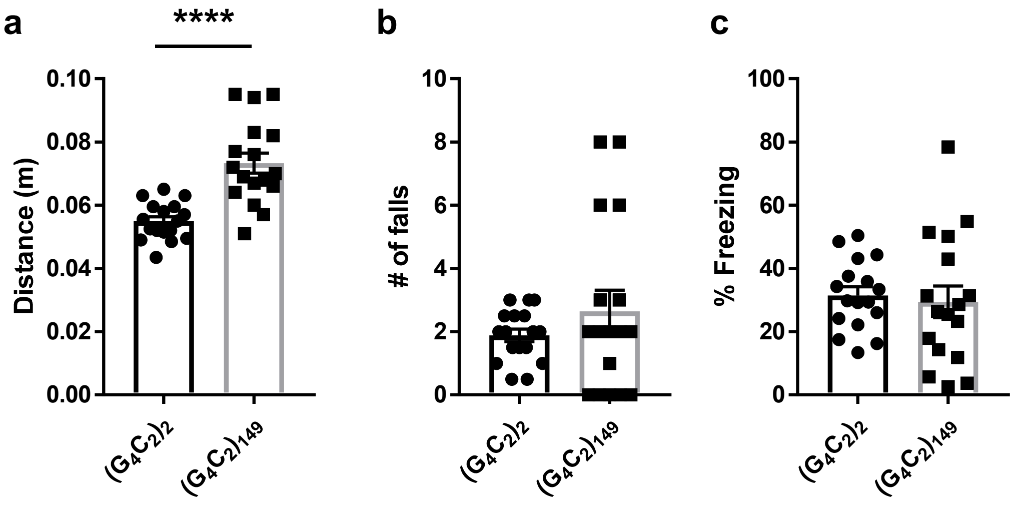

Figure S1. Hyperactivity precedes cognitive and motor deficits in $(G_4C_2)_{149}$–mice at 3 months of age. a) Significant increase in distance traveled in $(G_4C_2)_{149}$ mice in open field analysis indicative to hyperactivity. **b**) Hanging wire test revealed no difference in the number of falls in $(G_4C_2)_{149}$ mice relative to age-matched $(G_4C_2)_2$ mice. **c**) Contextual fear conditioning demonstrated no difference in freezing between $(G_4C_2)_2$ and $(G_4C_2)_{149}$ mice at 3 months of age. Data represent the mean ± SEM. ****p<0.0001 as analyzed by unpaired two-tailed t tests.

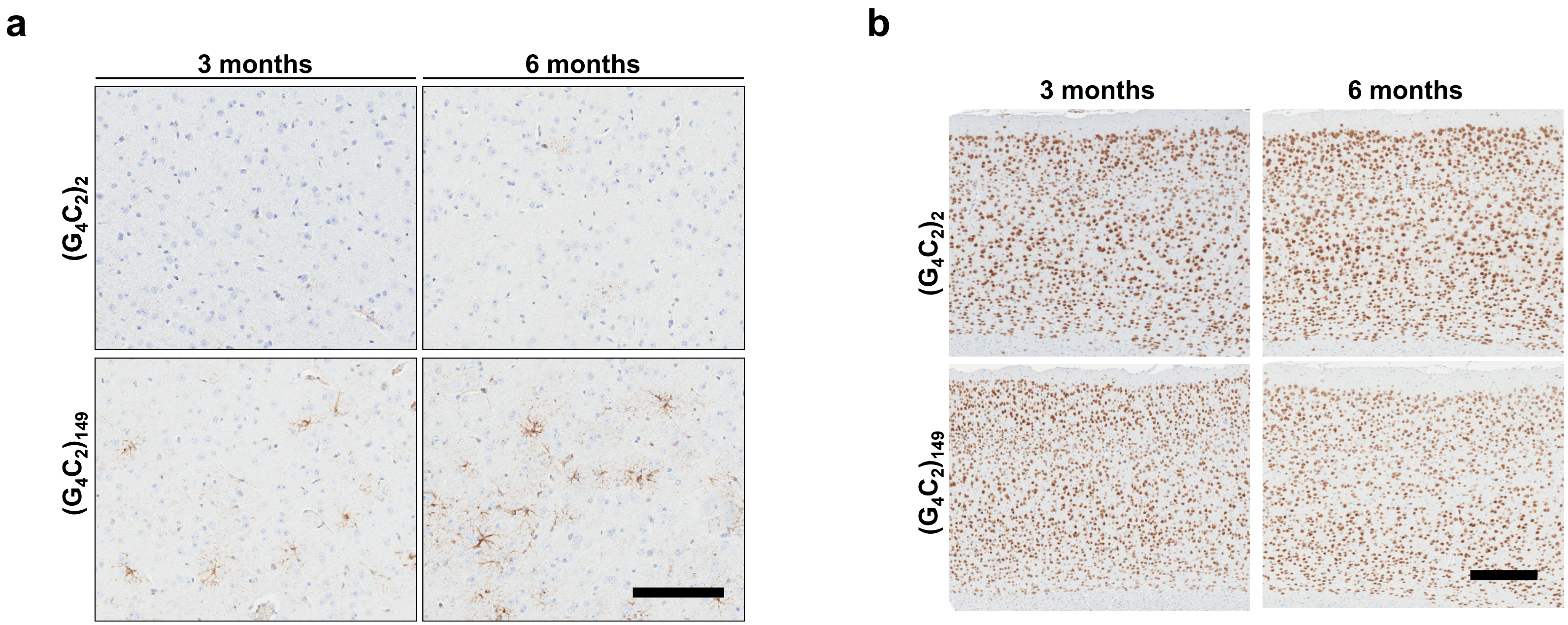

Figure S2. $(G_4C_2)_{149}$–mice exhibit gliosis and neurodegeneration at 6 months of age. a-b) Representative images of immunohistochemical analysis of GFAP (a) and NeuN (b) in the cortex of $(G_4C_2)_2$ and $(G_4C_2)_{149}$ mice. Scale bar represents 150μm.

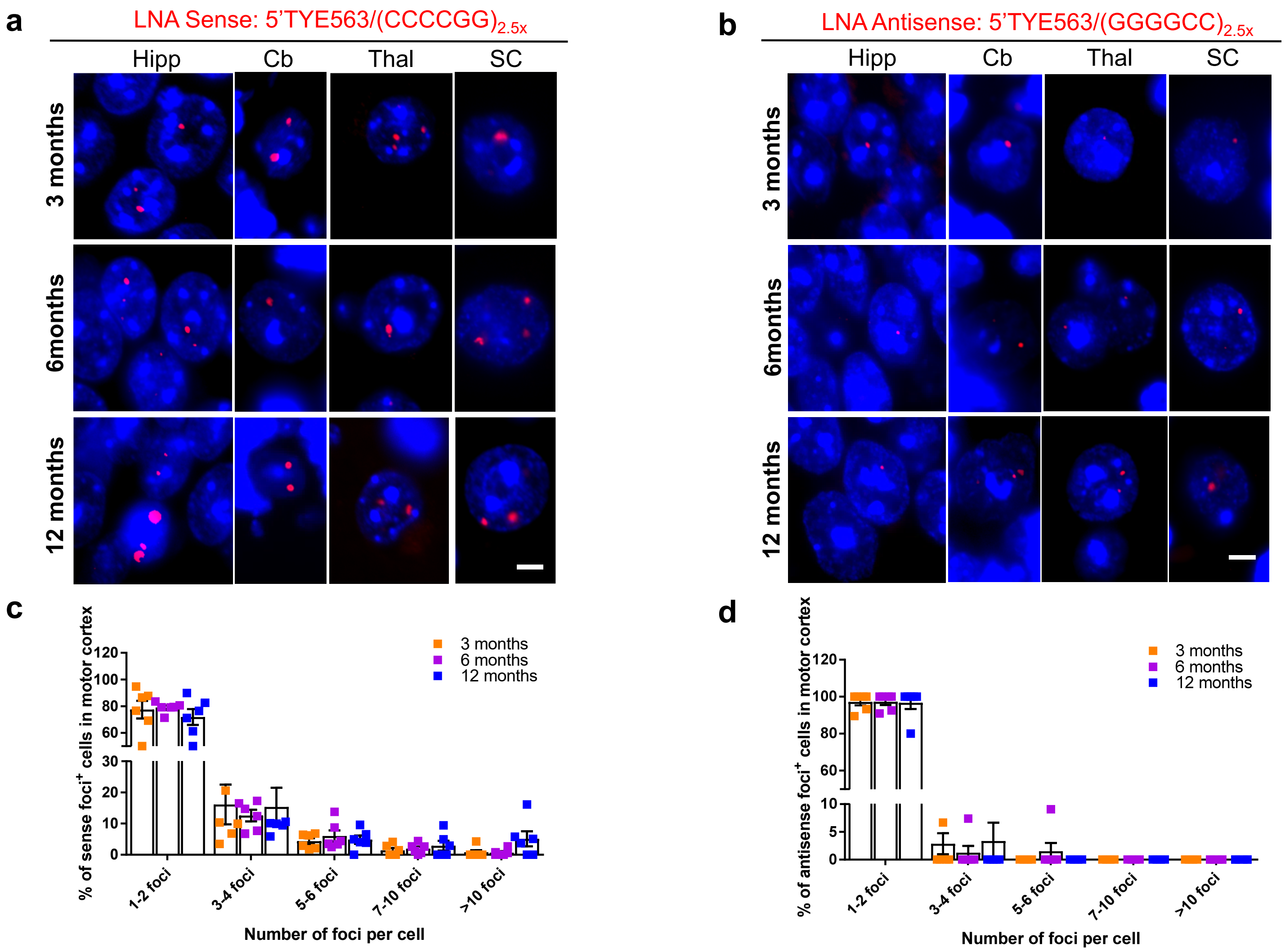

Figure S3. Sense and antisense RNA foci observed in the CNS of $(G_4C_2)_{149}$–mice. a-b) Sense (**a**) and antisense (**b**) were observed in the hippocampus, Purkinje layer of the cerebellum, thalamus, and anterior horn of the spinal cord. **c-d**) Quantitative analysis of the number of sense (**c**) or antisense (**d**) RNA foci present in foci-bearing cells in the motor cortex of $(G_4C_2)_{149}$–mice at 3, 6, and 12 months of age (n=6per age group). Scale bar represents 5μm.

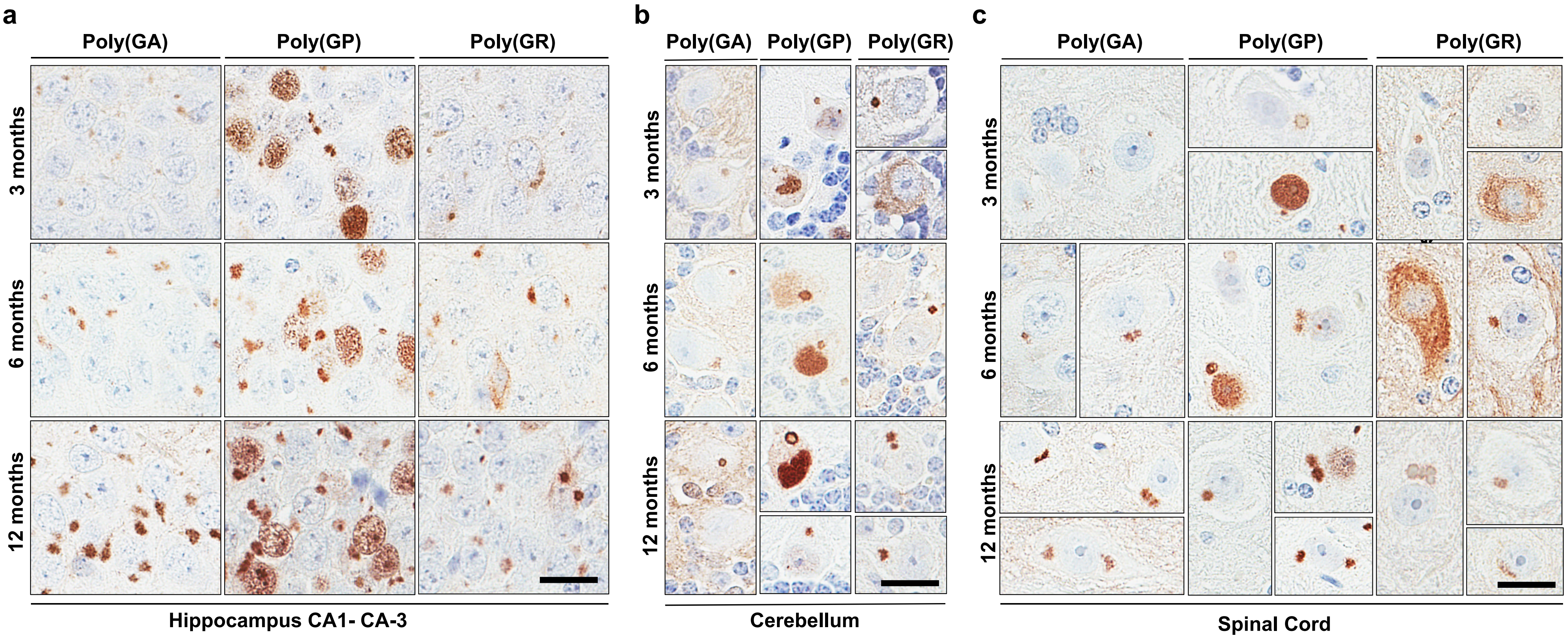

Figure S4. Sense DPR pathology detected throughout the CNS in $(G_4C_2)_{149}$–mice. a-c) Representative images of immunohistochemical analysis of poly(GA), poly(GP), and poly(GR) in the hippocampus (**a**), Purkinje layer of the cerebellum (**b**), and ventral horn of the spinal cord (**c**). Scale bar represents 20μm.

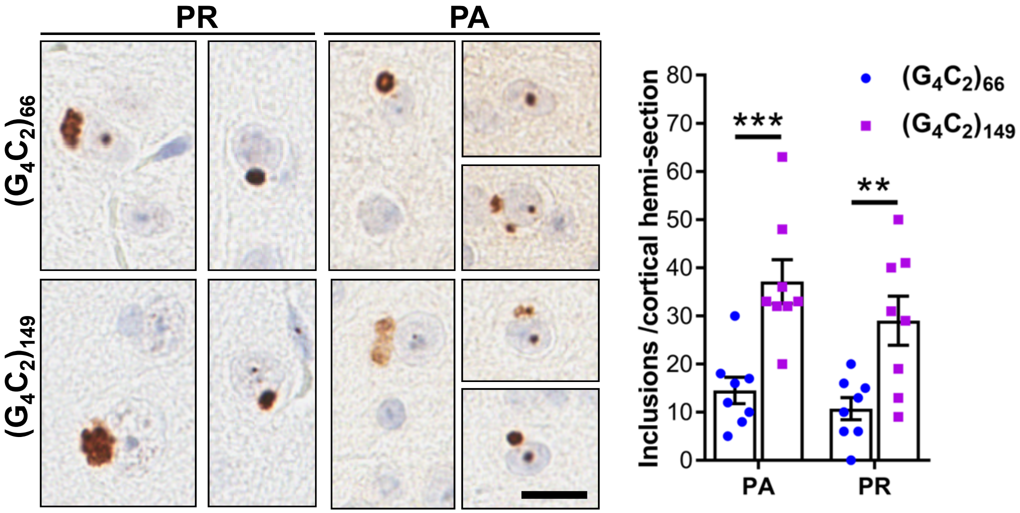

Figure S5. Antisense DPR pathology detected in both $(G_4C_2)_{66}$ and $(G_4C_2)_{149}$–mice. Representative images of immunohistochemical analysis and quantitation of poly(PR) and poly(PA) inclusions in the cortex of $(G_4C_2)_{66}$ and $(G_4C_2)_{149}$–mice at 6 months of age. Data represent the mean ± SEM. $^{**}p<0.01$ and $^{***}p<0.001$ as analyzed by unpaired two-tailed t tests. Scale bar represents 20μm.

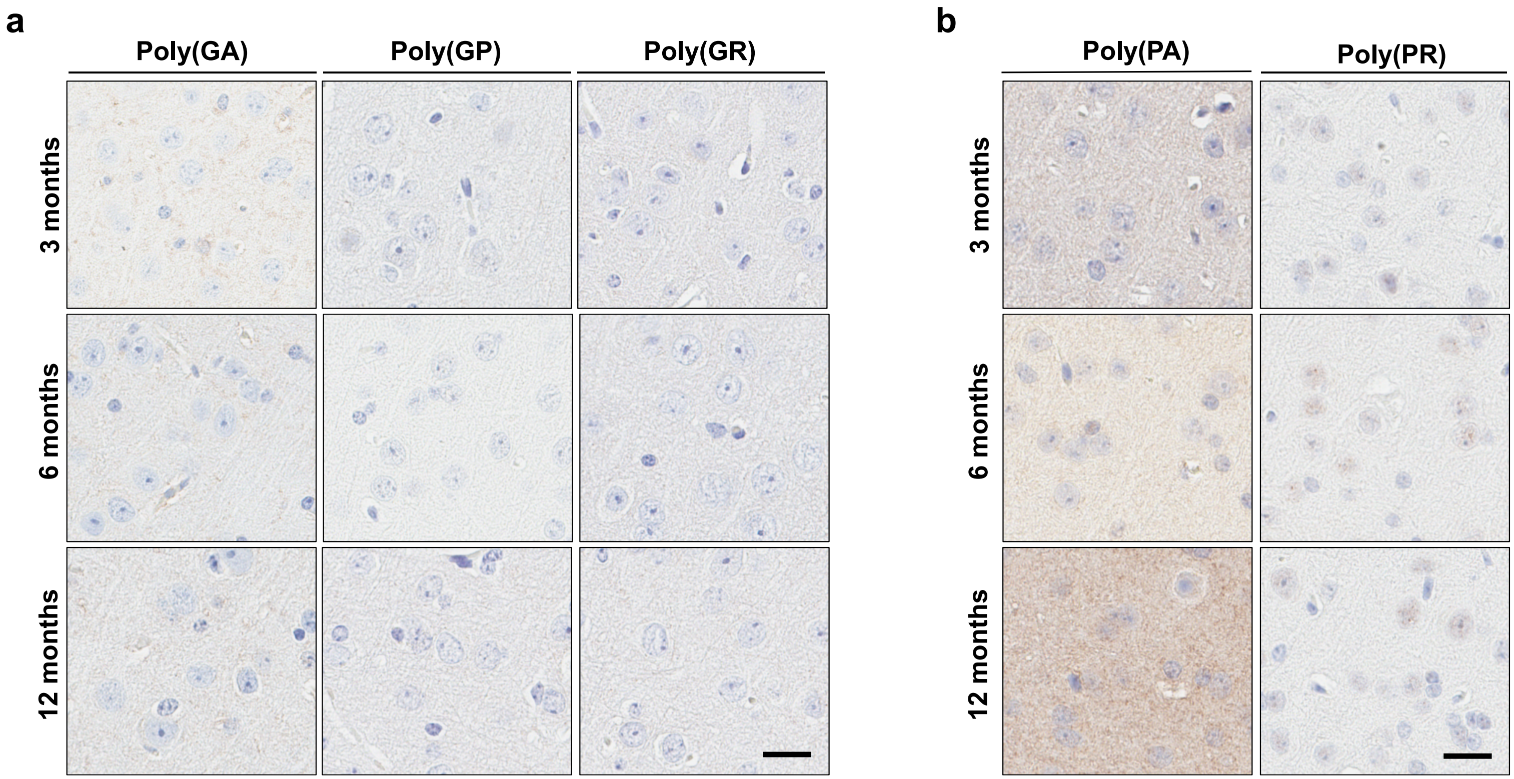

Figure S6. Sense and antisense DPR pathology is not detected in control $(G_4C_2)_2$–mice. a-b) Immunohistochemical analysis of poly(GA, poly(GP), poly(GR), poly(PA), and poly(PR) demonstrates absence of pathology in control $(G_4C_2)_2$ mice at all ages evaluated. Scale bar represents 20μm.

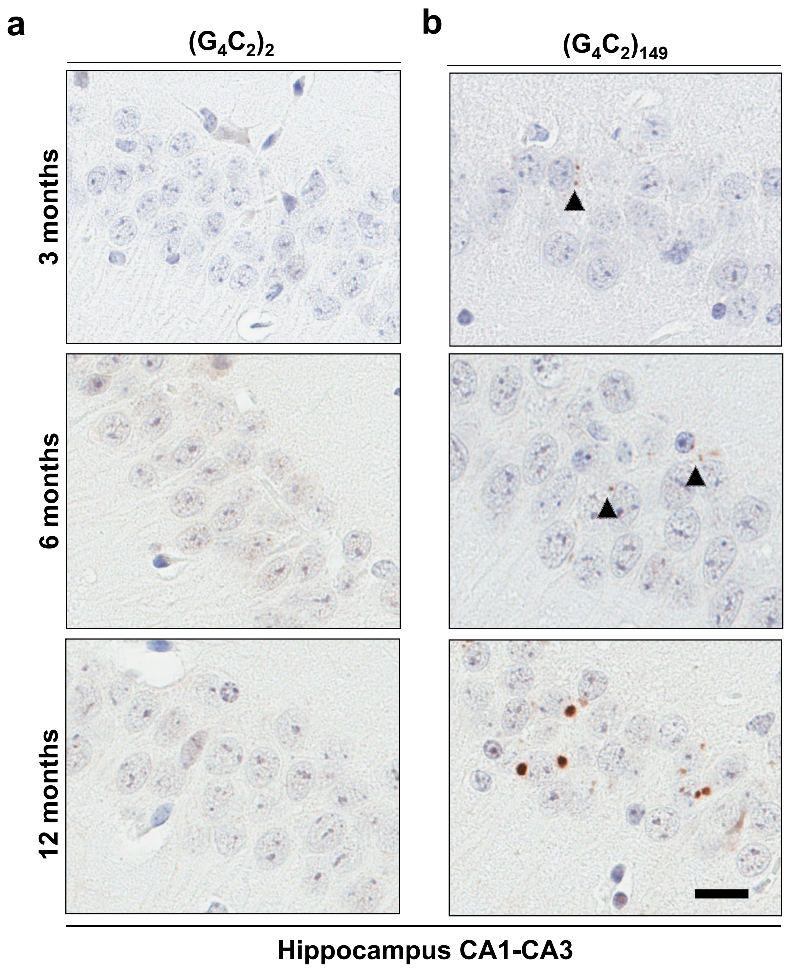

Figure S7. Hippocampal pTDP-43 (pS409/410) pathology detected in $(G_4C_2)_{149}$–mice. a-b) Representative images of immunohistochemical analysis of pTDP-43 in the hippocampus of $(G_4C_2)_2$ (**a**) and $(G_4C_2)_{149}$ mice (**b**) at 3, 6, and 12 months of age (inclusions indicated by black arrowheads). Scale bar represents 20μm.

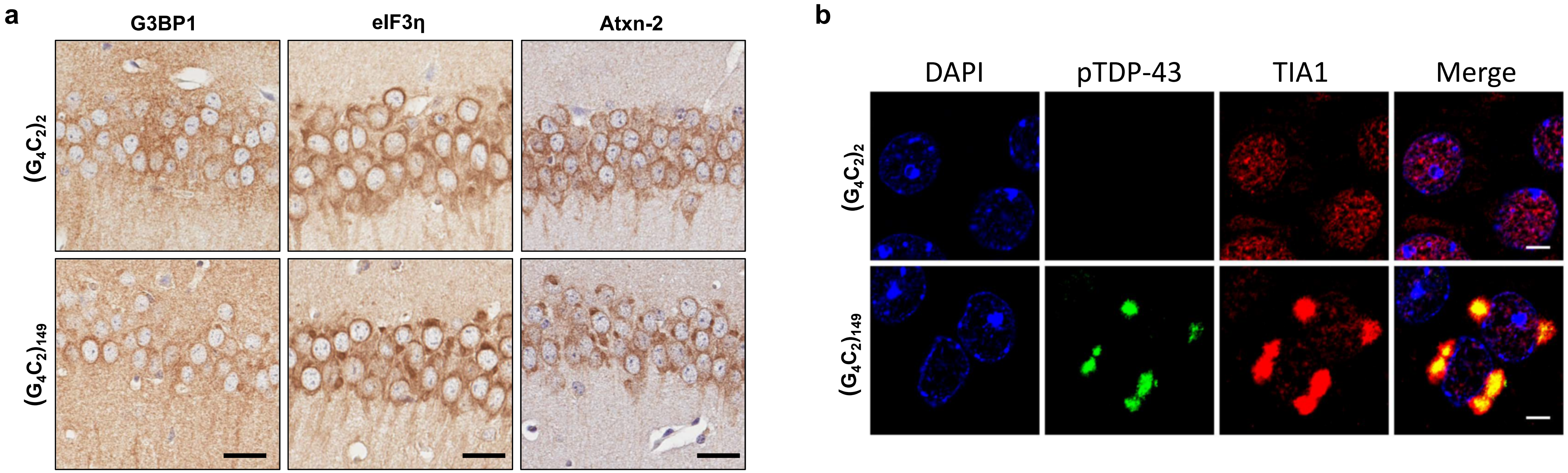

Figure S8. Deposition of stress granule-associated proteins in $(G_4C_2)_{149}$–mice. a) Representative images of immunohistochemical analysis of G3BP1, eIF3η, and ataxin-2 in the hippocampus of $(G_4C_2)_2$ and $(G_4C_2)_{149}$ mice at 12 months of age. **b**) Representative immunofluorescent images depicting colocalization between pTDP-43 (green) and TIA-1 (red) in $(G_4C_2)_{149}$ mice, with control $(G_4C_2)_2$ mice characterized by the absence of pTDP-43 pathology and TIA-1 exhibiting a normal nuclear distribution. Nuclei are labeled with DAPI. Scale bars represent 20µm (**a**) and 5µm (**b**).
